# Random Sampling with Interspike-Intervals of the Exponential Integrate and Fire Neuron: A Computational Interpretation of UP-States

**DOI:** 10.1371/journal.pone.0132906

**Published:** 2015-07-23

**Authors:** Andreas Steimer, Kaspar Schindler

**Affiliations:** Department of Neurology, Inselspital\Bern University Hospital\University Bern, Bern, Switzerland; McGill University, CANADA

## Abstract

Oscillations between high and low values of the membrane potential (UP and DOWN states respectively) are an ubiquitous feature of cortical neurons during slow wave sleep and anesthesia. Nevertheless, a surprisingly small number of quantitative studies have been conducted only that deal with this phenomenon’s implications for computation.

Here we present a novel theory that explains on a detailed mathematical level the computational benefits of UP states. The theory is based on random sampling by means of interspike intervals (ISIs) of the exponential integrate and fire (EIF) model neuron, such that each spike is considered a sample, whose analog value corresponds to the spike’s preceding ISI. As we show, the EIF’s exponential sodium current, that kicks in when balancing a noisy membrane potential around values close to the firing threshold, leads to a particularly simple, approximative relationship between the neuron’s ISI distribution and input current. Approximation quality depends on the frequency spectrum of the current and is improved upon increasing the voltage baseline towards threshold. Thus, the conceptually simpler leaky integrate and fire neuron that is missing such an additional current boost performs consistently worse than the EIF and does not improve when voltage baseline is increased. For the EIF in contrast, the presented mechanism is particularly effective in the high-conductance regime, which is a hallmark feature of UP-states.

Our theoretical results are confirmed by accompanying simulations, which were conducted for input currents of varying spectral composition. Moreover, we provide analytical estimations of the range of ISI distributions the EIF neuron can sample from at a given approximation level. Such samples may be considered by any algorithmic procedure that is based on random sampling, such as Markov Chain Monte Carlo or message-passing methods.

Finally, we explain how spike-based random sampling relates to existing computational theories about UP states during slow wave sleep and present possible extensions of the model in the context of spike-frequency adaptation.

## 1 Introduction

Since the time of its earliest discovery [1; 2], the transient dynamics of a neuron’s baseline membrane potential between high values close to the firing threshold (UP-state) and low levels close to the resting potential (DOWN-state), both during slow-wave sleep and anesthesia, have received considerable attention. Despite the large amount of work that has been spent on the genesis of this phenomenon (see e.g. [3; 4; 5; 6; 7; 8; 9; 10]), only comparably few studies have implicitly or explicitly dealt with its implications for information processing and computation [11; 12; 13; 14; 7; 15]. Although amongst the latter a variety of qualitative theories has been pushed forward, such as reliable information storage [7], the sustaining of activity for a neural representation of working memory [11] or memory consolidation during slow wave sleep [12; 13], even fewer quantitative computational theories about the role of transient UP and DOWN-states have been formulated [14; 15].

On the other hand, separate lines of research have indicated that interspike intervals (ISIs), i.e. the time-lags between two successive action potentials, may contain information valuable for computation, both across species and sensory modalities [16; 17; 18; 19; 20]. In [16] for example, stimulus information contained by spikes from neurons in area V1 was shown to depend strongly on a spikes preceding ISI. Furthermore, the structure of log-ISI histograms obtained from such neurons was found impossible to be explained on the basis of rate modulations alone (see also [20]).

**Fig 1:**
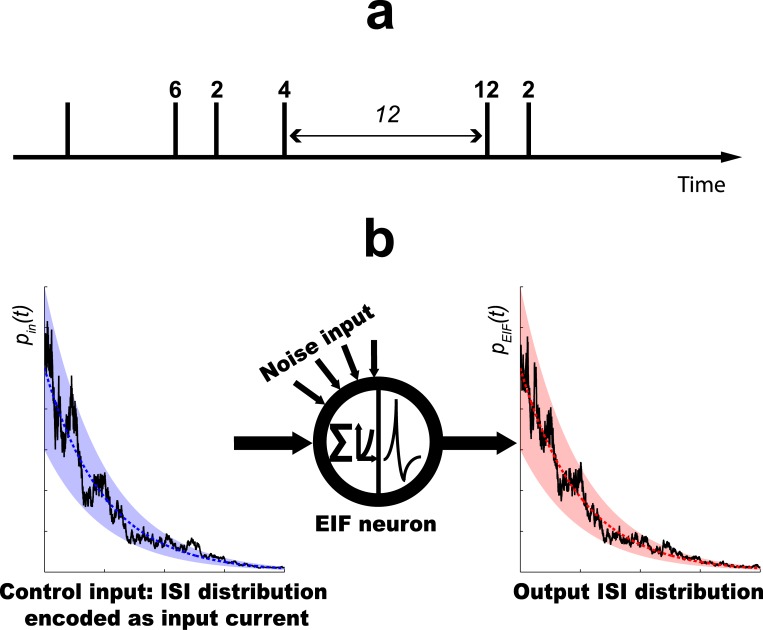
Basic Principles of the Proposed Model. a) Random sampling with interspike intervals (ISIs): Each spike in a train is interpreted as carrying an analog label, whose value corresponds to the length of the ISI preceding the spike, i.e. to the difference in spike times between the considered spike and its predecessor (see numbers above spikes in arbitrary units). These analog values are therefore samples from the ISI distribution *p*(*t*) underlying the spike train. Depending on the computational context, *p*(*t*) may be stationary or not. (Figure adapted with permission from [25]) b) EIF neuron as ISI sampler and probability transducer: A ‘user-defined’, target ISI distribution *p*_*in*_(*t*) that is given by a small, multiplicative modulation (left black trace) of an exponential distribution (blue/red dotted trace) is specified as input current to the EIF neuron. Subject to noise, the neuron responds with a membrane potential fluctuating around some baseline value and thus with the stochastic firing of output spikes, whose ISI distribution also follows a modulated exponential distribution *p*_*EIF*_(*t*) (right black trace). Our theory shows that modulations at the output may approximate those at the input, provided the latter stay within sufficiently small margins (blue/red shaded areas). Approximations are improved upon increasing the baseline level of the membrane potential towards firing threshold.

Based on the exponential integrate and fire (EIF) neuron model [21], we here present a computational interpretation of UP-states, which matches in a detailed, quantitative way the functional properties of the EIF neuron with computational requirements of an ideal ISI-based random sampler. More specifically, we show how the sequence of ISIs, elicited by a current driven EIF neuron during UP-states, may correspond to a sequence of random numbers (Fig 1a), that can be utilized within any algorithmic procedure that is based on random sampling, such as Markov-Chain-Monte-Carlo (MCMC)-methods [22; 23], or message-passing algorithms [24; 25]. At its core, our method relies on an approximative analytic matching between the ISI distribution of the EIF and an ‘ideal ISI-sampler’, whose firing is controlled by standard equations from renewal theory. The ISI distribution the neuron is supposed to sample from may be defined as a multiplicative modulation of an exponential distribution and is specified by the input current (Fig 1b). As we show, for the EIF neuron but not the ordinary leaky integrate and fire (LIF) neuron, this approximation depends on the baseline level of the membrane potential and improves upon increasing this level towards firing threshold. This way our theory provides a computational meaning to UP-states.

This paper is organized as follows: After providing the necessary fundamentals of renewal theory and the EIF/LIF model, we analytically derive a match between the two and the approximations/assumptions it relies on. By analyzing this situation in the frequency domain (spectral analysis), performance of the LIF neuron is shown to be consistently worse compared to the EIF. These theoretical results are then corroborated by accompanying simulations, where the two types of neurons are used to sample from ISI distributions of varying spectral composition. Subject to the assumptions behind our analytic derivation, an estimation of the range of ISI distributions from which the EIF neuron can sample is subsequently derived. In the discussion part, we qualitatively describe implications of the model for interpreting spike data from experiments, along with a possible extension of the model that includes spike-frequency adaptation. Model predictions and a more computational interpretation of the meaning of ISI-based random sampling during slow-wave sleep are also provided.

## 2 Methods

### 2.1 Fundamental Dependencies of Renewal Theory

Here we briefly present some basic equations from the field of renewal theory, which relate two quantities that are key to the contents of this paper: The stochastic intensity (or hazard *h*) and the interspike interval distribution *p*.

Intuitively the hazard can be regarded as a conditional instantaneous firing rate (i.e. *h*(*t*)*dt* gives the probability of firing a spike in an infinitesimally small interval *dt* around *t*), where the conditioning is on the time of the last spike. In other words, *h*(*t*) is a time dependent rate profile that is not influenced in any way by spiking activity prior to the last spike. Assuming the last spike to have happened at time *t*′ = 0, a standard result from renewal theory relates *h*(*t*) to *p*(*t*) in the following way [26; 27]:

p(t)=S(t)h(t) (2.1)

$$S\left(t\right)≔1-\int_{0}^{t}\;p\left(t^{\prime }\right)dt^{\prime }=\exp(-\int_{0}^{t}\;h\left(t^{\prime }\right)dt^{\prime })$$ (2.2)

where *S*(*t*) gives the probability of not firing (’surviving’) until time *t* and is hence called the survivor function. Eq 2.1 has a simple, intuitive interpretation: The probability of an ISI of length *t* is equal to the probability of not firing until *t* (*S*(*t*)), times the probability of a spike exactly at *t*, given that the neuron has not fired so far (*h*(*t*)). Conversely, Eq 2.1 may be solved for *h*(*t*), thereby expressing the hazard in terms of *p*(*t*) and the survivor function

$$h\left(t\right)=p\left(t\right)\exp(\int_{0}^{t}\;h\left(t^{\prime }\right)dt^{\prime })$$ (2.3)

$$\Rightarrow \frac{d}{dt}\text{ln}\;h\left(t\right)-h\left(t\right)=\frac{d}{dt}\text{ln}\;p\left(t\right)$$ (2.4)

It is differential Eq 2.4 that is followed by what we define as an ideal ISI sampler: If the term on the right hand side is interpreted as a time dependent input to the sampler (e.g. a current injected into a neuron) and the samplers hazard dynamics are guaranteed to follow 2.4, then its output ISI distribution will be directly determined by the input. In other words, the input-specified ISI distribution will be transduced to the samplers output without distortion (cf.Fig 1b). As we show, Eq 2.4 can be approximated by an EIF neuron and we will thus call it *the hazard equation of the ideal ISI-sampler*.

### 2.2 The Exponential Integrate-and-Fire Model Combined with Stochastic Firing

In the EIF model, a neuron’s membrane potential dynamics are given by the following differential equation [21]

$$C_{m}\dot{V}\left(t\right)=-g_{L}\left(V\left(t\right)-E_{L}\right)+g_{L}\Delta_{T}\exp(\frac{V\left(t\right)-V_{T}}{\Delta_{T}})+I\left(t\right)$$ (2.5)

$$\Leftrightarrow \;\;\;\;\;\;\dot{V}\left(t\right)=-\frac{1}{\tau_{m}}\left(V\left(t\right)-E_{L}\right)+\frac{1}{\tau_{m}}\Delta_{T}\exp(\frac{V\left(t\right)-V_{T}}{\Delta_{T}})+I^{*}\left(t\right)$$ (2.6)

where *V* is the membrane potential, $\tau_{m}≔\frac{C_{m}}{g_{L}}$ the membrane time constant based on leak conductance *g*_*L*_ and membrane capacitance *C*_*m*_. $I^{*}≔\frac{I}{C_{m}}$ is the total input current of the neuron divided by the membrane capacitance, *E*_*L*_ the leak reversal potential, *V*_*T*_ the threshold voltage and Δ_*T*_ the so called slope factor. The slope factor determines the effectiveness of the exponential term in Eq 2.6, which mimics the continuously increasing number of open sodium channels when *V* approaches the threshold *V*_*T*_ and consequently leads to a strong increase of current into the neuron. Once *V*_*T*_ is crossed, positive feedback induced by the exponential term renders the voltage dynamics self-sustained. That is, even in the absence of any driving current *I*, *V* starts to diverge to infinity in finite time. Therefore, a spike is said to have occurred once *V* crosses a peak potential *V*_*p*_ ≥ *V*_*T*_, after which *V* is reset to some reset potential *V*_*r*_ (see methods section 2.5 for a list of parameter values that we have used for the simulations presented in the results section). Note that the thus defined dynamics of the EIF neuron contain those of the more conventional LIF neuron as a special case, namely if *V*_*p*_ = *V*_*t*_ and Δ_*T*_ → 0.

For the EIF neuron, the voltage trajectory between the times at which *V*_*T*_ and *V*_*p*_ were respectively crossed is supposed to qualitatively match the spike waveform of biological neurons. Importantly however, the opening of sodium channels influences voltage dynamics even below the threshold, which, as we explain in the results section, is crucial for a neural approximation of the hazard equation of the ideal ISI-sampler (Eq 2.6). For the same reason, we make use of the high-conductance regime, consisting of a large *g*_*L*_ and, consequently, a small *τ*_*m*_. Interestingly, it is also this regime that is characteristic for UP-states [28; 29; 30] (More specifically, it is the 3–5 fold increase in *synaptic*, not *leak* conductance that is characteristic for UP-states. However, based on the Ansatz in [31], the two types of synaptic synaptic conductances (exc./inh.) can be rearranged, such that their (constant) average plays the role of a leak conductance. This way, Eq 2.5 is reobtained with a higher leak conductance and new *V*_*T*_, *I*(*t*), both of which were redefined through the addition of constants).

In addition, for the approach put forward in this paper, the EIF/LIF voltage dynamics of Eq 2.6 are combined with a stochastic firing criterion, such that spikes may be fired even below the threshold. More specifically, we introduce a firing hazard, which depends exponentially on the membrane potential trajectory via some convolution kernel 𝓚(*t*) that is normalized to $\int_{-\infty }^{\infty }𝓚(t)\text{d}t=1$.

$$h\left(t\right)≔\frac{1}{K\tau_{m}}\exp(\frac{(𝓚*\;(V-V_{T}))\;\left(t\right)}{\Delta_{T}})$$ (2.7)

where *K* is a dimensionless scaling factor for adjusting the max. firing rate of the neuron at threshold (which we have chosen to be 10 Hz, based on the reported, low values for the firing rate during UP-states, see e.g. [3; 32]). Note that in Eq 2.7 the spiking determinism parameter is assumed to be identical to the slope-factor Δ_*T*_ = 3mV of the EIF neuron, both for the EIF and LIF neuron. In other words, throughout the manuscript we have exclusively used in Eq 2.7 a spiking determinism parameter equal to 3mV, although in case of the LIF neuron the slope factor controlling membrane potential dynamics was zero. This way both types of neurons may be compared on a fair basis, with all factors being equal except the slope factor.

The hazard *h*(*t*) in Eq 2.7 is an abstraction of what is called diffusive noise [27], i.e. the strong voltage fluctuations measured in biological neurons, due to their random bombardment by a large number of balanced inhibitory and excitatory inputs. In particular, such a network effect is thought to be a characterizing feature of UP-states [33; 3; 34; 5; 4; 8]. Whereas the hazard Eq 2.7 involves a deterministic membrane potential trajectory combined with stochastic firing, diffusive noise is based on a noisy membrane potential combined with deterministic firing (a spike is fired only if a fixed threshold is crossed). Although intuitively the two noise models may seem to be equivalent, in general they are not, but may well approximate each other phenomenologically in case of the more widespread LIF neuron [35]. For the EIF neuron however, no such phenomenological model exists so far, which is why for this study we have used the hazard Eq 2.7 as a proxy for the biologically more realistic case of diffusive noise. Note however that Eq 2.7 only assumes the logarithm of the hazard *h*(*t*) to depend on the membrane potential trajectory *V*(*t*) via some linear filter (with normalized filter kernel), an assumption that entails the frequently used exponential escape noise model [27] as a special case, i.e. when 𝓚 is set to 𝓚(*t*) = *δ*(*t*) such that for any time *t* the hazard depends only on the momentary distance between *V*(*t*) and *V*_*T*_. If 𝓚(*t*) is not normalized to 1 the normalization constant may be absorbed by Δ_*T*_, provided the normalization constant is positive.

### 2.3 Construction and Performance Evaluation of Example ISI Distributions

We evaluated the representational capabilities of the EIF and LIF neuron based on two types of target ISI distributions *p*_*in*_(*t*) = *p*_0_(*t*)Δ*p*_*in*_(*t*), where *p*_0_(*t*) ≔ *h*_0_exp(−*h*_0_ *t*) is an exponential ‘baseline’ distribution with hazard *h*_0_ and Δ*p*_*in*_(*t*) a modulation function ‘around’ *p*_0_(*t*) (e.g. multiplicative noise).

For the first type of target distribution (low-frequency noise), ln Δ*p*_*in*_(*t*) was chosen as a superposition of 60 sinusoids with random phases and unit amplitudes. Frequencies of the sinusoids were taken equidistantly from the interval [10, 100] Hz. The thus obtained ln Δ*p*_*in*_(*t*) was then centered to a mean of zero and scaled such that the min/max range of Δ*p*_*in*_(*t*) was 1 ± *r*_Δ*p*_. The parameter 0 ≤ *r*_Δ*p*_ ≤ 1 controls the degree by which the (unnormalized) *p*_*in*_(*t*) deviates from *p*_0_(*t*) and we will refer to it as the probability fluctuation ratio. The second type of modulation function (high-frequency noise) was constructed in an identical manner, but with frequencies chosen from [100, 200] Hz. Finally, both types of modulation functions were multiplied by a constant, such that the resulting *p*_*in*_(*t*) was normalized to one.

In both cases, we systematically varied *h*_0_ and *r*_Δ*p*_ and, for each such combination, evaluated the quality of the EIF and LIF neuron to approximate Δ*p*_*in*_(*t*). For that, $p_{\overset{\text{EIF;}}{LIF}}\left(t\right)≔p_{0}\left(t\right)\Delta p_{\overset{\text{EIF;}}{LIF}}\left(t\right)$ -the output ISI distributions of the EIF and LIF neuron respectively- were obtained by numerical integration of Eq 2.1, based on the neuron’s membrane potential *V*(*t*) and Eq 2.7. *V*(*t*) was given in response to some input current *I*(*t*), which was suitably chosen in order for the neuron to approximate the target probability modulation Δ*p*_*in*_(*t*) (see results). To produce a ‘clean’ membrane potential trajectory suitable for numerical integration, the neuron was prevented from firing in the subthreshold region, but could fire and reset once the threshold (LIF case), or the peak potential (EIF case) had been crossed.

The neuron’s performance of approximating ln Δ*p*_*in*_(*t*) was evaluated using the normalized *L*1-distance:

$$L1_{norm}\left(\text{ln}\Delta p_{in},\text{ln}\Delta p_{EIF;LIF}\right)≔\frac{{\left||\text{ln}\Delta p_{in}-\text{ln}\Delta p_{EIF;LIF}|\right|}_{1}}{{\left||\text{ln}\Delta p_{in}|\right|}_{1}+{\left||\text{ln}\Delta p_{EIF;LIF}|\right|}_{1}}$$ (2.8)

$${\left||f|\right|}_{1}≔\int_{0}^{\infty }|f(t)|dt$$ (2.9)

### 2.4 Analytic Derivation of the Probability Modulation Transfer Function

Here we give full account of how the probability modulation transfer function (Eq 3.20) is derived, as it is central to our spectral analysis. In the following, we restrict ourself to the EIF neuron, such that cluttered notation is avoided. The LIF case may be derived analogously.

Assume the multiplicative probability modulation Δ*p*_*EIF*_(*t*) to be caused by some additive, small hazard modulation Δ*h*(*t*). Then, according to Eqs 2.4 and 3.16

$$\frac{d}{dt}\text{ln}\left(h_{0}+\Delta h\left(t\right)\right)-\left(h_{0}+\Delta h\left(t\right)\right)=\frac{d}{dt}\text{ln}\;p_{EIF}\left(t\right)=\frac{d}{dt}\text{ln}\left(p_{0}\left(t\right)\Delta p_{EIF}\left(t\right)\right)$$ (2.10)

$$\Rightarrow \;\;\;\;\;\frac{d}{dt}\text{ln}\;(1+\frac{\Delta h(t)}{h_{0}})-\Delta h\left(t\right)=\frac{d}{dt}\text{ln}\Delta p_{EIF}\left(t\right)$$ (2.11)

If Δ*h*(*t*)/*h*_0_ < < 1, then $\text{ln}\;\left(1+\frac{\Delta h(t)}{h_{0}}\right)\approx \frac{\Delta h(t)}{h_{0}}$ and hence

$$\frac{1}{h_{0}}\frac{d}{dt}\Delta h\left(t\right)-\Delta h\left(t\right)=\frac{d}{dt}\text{ln}\Delta p_{EIF}\left(t\right)$$ (2.12)

Assuming Δ*h*(0) = ln Δ*p*_*EIF*_(0) = ln Δ*p*_*in*_(0) = 0, Laplace transformation of Eq 2.12 then yields the intermediate result

$$𝓛\{\Delta h(t)\}\left(s\right)=\frac{h_{0}s}{s-h_{0}}𝓛\{\text{ln}\Delta p_{EIF}\left(t\right)\}\left(s\right)$$ (2.13)

On the other hand, following the derivations of section 3.1, we know that for the EIF neuron ln *p*_*EIF*_(*t*) = ln (*p*_0_(*t*)Δ*p*_*EIF*_(*t*)) develops according to Eq 3.14 and hence

$$\frac{d}{dt}\text{ln}\Delta p_{EIF}\left(t\right)=\frac{d}{dt}\text{ln}\;p_{EIF}\left(t\right)-\frac{d}{dt}\text{ln}\;p_{0}\left(t\right)=K_{EIF,0}\;\Delta h\left(t\right)+\frac{d}{dt}\text{ln}\Delta p_{in}\left(t\right)$$ (2.14)

which upon the Laplace transform gives

$$s\cdot 𝓛\{\text{ln}\Delta p_{EIF}\left(t\right)\}\left(s\right)=K_{EIF,0}\;𝓛\{\Delta h(t)\}\left(s\right)+s\cdot 𝓛\{\text{ln}\Delta p_{in}\left(t\right)\}\left(s\right)$$ (2.15)

Inserting the intermediate result Eq 2.13 and rearranging terms then yields the probability modulation transfer function Eq 3.20.

### 2.5 Parameters used in Numerical Simulations and Spectral Analysis

Table 1 lists all parameters and values we have used for our numerical simulations of the EIF- and LIF-neuron and for obtaining results of the spectral analysis of section 3.2.

**Table 1:** Parameters underlying the numerical simulations of the EIF/LIF neuron and the theoretical results of section 3.2.

Parameter	Description	Value	Remarks
*dt*	Simulation time step	0.05 ms	Used by a 3^*rd*^ order Runge-Kutta method for num. integration
*V*_*T*_	Threshold potential	-50.4 mV	
*V*_*p*_	Peak potential	-40.4 mV	
*V*_*r*_	Reset potential	*V*_0_	*V*_*r*_ differed for each voltage/hazard baseline, mimicking the small effect of AHP on membrane voltage in cortical regular spiking neurons during UP-states, see e.g. [5; 8]
*E*_*L*_	Leak reversal potential	-70.6 mV	
Δ_*T*_	Slope-factor	3 (0) mV	For the EIF (LIF) neuron respectively. In both cases Δ_*T*_ = 3ms was used for the hazard model of Eq 2.7
*C*_*m*_	Membrane capacitance	0.281 nF	
*g*_*L*_	Membrane leak conductance	5·30 nS (1·30 nS)	In the high (low) conductance regime respectively
*τ*_*m*_	Membrane time constant	$\frac{C_{m}}{g_{L}}\approx 1.9(9.4)$ ms	In the high (low) conductance regime respectively
*K*	Hazard scaling factor	$\frac{1}{10Hz\cdot \tau_{m}}\approx 53.4(10.7)$	In the high (low) conductance regime respectively

## 3 Results

### 3.1 Mapping the EIF Voltage Dynamics onto the Hazard Equation of the Ideal ISI-Sampler

Our first result is a mathematical description of how EIF voltage dynamics (Eq 2.6), together with the hazard model of Eq 2.7, enable the neuron to fire with an ISI distribution *p*_*EIF*_(*t*) ≔ *p*_0_(*t*)Δ*p*_*EIF*_(*t*) that is given by the product of a baseline exponential distribution *p*_0_(*t*) ≔ *h*_0_ exp (−*h*_0_ *t*) and some controllable modulatory term Δ*p*_*EIF*_(*t*) > 0. To achieve controllability, as we will show, the neuron’s input current I*(*t*) is supposed to encode for some ‘user-defined’ distribution *p*_*in*_(*t*) ≔ *p*_0_(*t*)Δ*p*_*in*_(*t*) > 0, such that under distinct conditions Δ*p*_*EIF*_(*t*) ≈ Δ*p*_*in*_(*t*) (see Fig 1b). For the LIF neuron however, the analogously defined *p*_*LIF*_(*t*) ≔ *p*_0_(*t*)Δ*p*_*LIF*_(*t*) leads to an approximation Δ*p*_*LIF*_(*t*) ≈ Δ*p*_*in*_(*t*) that is consistently worse when compared to the EIF neuron.

**Fig 2:**
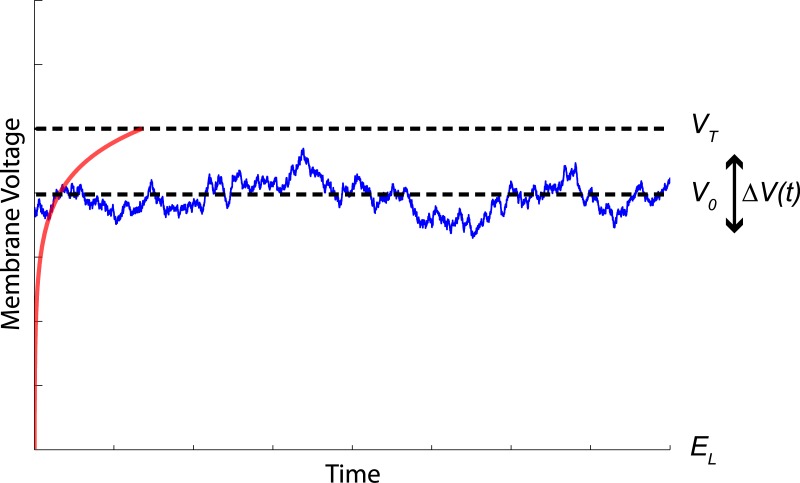
Dynamical Situation of the Membrane Potential During UP States of the EIF Neuron: Our approach assumes the membrane potential *V*(*t*) = *V*_0_ + Δ*V*(*t*) (blue trace) to fluctuate around some constant value *V*_0_, with time-dependent fluctuations Δ*V*(*t*). *V*_0_ is supposed to be close to the firing threshold *V*_*T*_ (and generally far from the resting (leak reversal) potential *E*_*L*_), such that the exponential input current of the EIF (red trace) becomes effective. The exponential term is crucial for an approximation of the ideal ISI sampler (see main text for details).

To mimic the voltage situation encountered during UP-states (Fig 2), the neuron’s membrane potential *V*(*t*) = *V*_0_ + Δ*V*(*t*) is assumed to fluctuate with some time-dependent voltage Δ*V*(*t*) around a constant baseline value *V*_0_ < *V*_*T*_, causing the hazard *h*(*t*) = *h*_0_ + Δ*h*(*t*) to fluctuate as well around *h*_0_. It then follows from Eq 2.7:

$$\Delta_{T}\text{ln}\frac{h(t)}{h_{0}}=(𝓚*\Delta V)\left(t\right)\text{,}\;\;\;\text{with}\;h_{0}≔\frac{1}{K\tau_{m}}\exp(\frac{V_{0}-V_{T}}{\Delta_{T}})$$ (3.1)

$$\Rightarrow \;\;\frac{d}{dt}(\Delta_{T}\text{ln}\frac{h(t)}{h_{0}})=(𝓚*\dot{\Delta V})\;\left(t\right)=(𝓚*\dot{V})\;\left(t\right)$$ (3.2)

$$\begin{array}{cc} & \overset{\text{(}2.6\text{)}}{=}\left(𝓚\;*\;\left(-\frac{1}{\tau_{m}}\left(V-E_{L}\right)+\frac{1}{\tau_{m}}\Delta_{T}\exp\;\left(\frac{V-V_{T}}{\Delta_{T}}\right)+I^{*}\left(t\right)\right)\right)\;\left(t\right)\\ & =-\frac{1}{\tau_{m}}\left(V_{0}-E_{L}\right)-\frac{1}{\tau_{m}}\left(𝓚*\Delta V\right)\left(t\right)+\frac{1}{\tau_{m}}\Delta_{T}\exp\;\left(\frac{V_{0}-V_{T}}{\Delta_{T}}\right). \end{array}$$ (3.3)

$$\left(𝓚*\exp\left(\frac{\Delta V}{\Delta_{T}}\right)\right)\;\left(t\right)+\left(𝓚*\;I^{*}\right)\left(t\right)$$ (3.4)

We now assume the fluctuations Δ*V*(*t*) to be sufficiently small such that:

(a) ∣Δ*V*(*t*)∣ ≪ Δ_*T*_
(b) ∣(𝓚*Δ*V*)(*t*)∣ ≪ Δ_*T*_
(c) $\frac{h(t)}{h_{0}}=1+\frac{\Delta h(t)}{h_{0}}\approx 1$

from which it follows

$$(𝓚*\exp(\frac{\Delta V}{\Delta_{T}}))\;\left(t\right)\overset{\text{(a)}}{\approx }(𝓚*\;(1+\frac{\Delta V}{\Delta_{T}}))\;\left(t\right)$$ (3.5)

$$=1+\frac{1}{\Delta_{T}}(𝓚*\Delta V)\;\left(t\right)$$ (3.6)

$$\overset{\text{(b)}}{\approx }\exp(\frac{1}{\Delta_{T}}(𝓚*\Delta V)\;\left(t\right))$$ (3.7)

$$\overset{\text{(}3.1\text{)}}{=}\;\frac{h(t)}{h_{0}}$$ (3.8)

where the approximations are due to the Taylor expansion of the exponential function around Δ*V* = 0. Subsequently, inserting Eqs 3.1 and 3.8 into Eq 3.4 yields

$$\Delta_{T}\frac{d}{dt}\text{ln}\frac{h(t)}{h_{0}}\approx -\frac{\Delta_{T}}{\tau_{m}}\text{ln}\frac{h(t)}{h_{0}}+\frac{\Delta_{T}}{\tau_{m}}\exp(\frac{V_{0}-V_{T}}{\Delta_{T}})\cdot \frac{h(t)}{h_{0}}+\left(𝓚*\;I^{*}\right)\left(t\right)-\frac{1}{\tau_{m}}\left(V_{0}-E_{L}\right)$$ (3.9)

$$\overset{\text{(c)}}{\approx }-\frac{\Delta_{T}}{\tau_{m}}\frac{\Delta h(t)}{h_{0}}+\frac{\Delta_{T}}{\tau_{m}}\exp(\frac{V_{0}-V_{T}}{\Delta_{T}})\cdot \frac{h(t)}{h_{0}}+\left(𝓚*\;I^{*}\right)\left(t\right)-\frac{1}{\tau_{m}}\left(V_{0}-E_{L}\right)$$ (3.10)

$$\Leftrightarrow \;\frac{d}{dt}\text{ln}\;h\left(t\right)\;\;\;\;\;\approx -Kc_{0}\cdot \Delta h\left(t\right)+K\cdot h\left(t\right)-\left(K-1\right)h_{0}+\frac{d}{dt}\text{ln}\;p_{in}\left(t\right)$$ (3.11)

where

$$c_{0}≔\exp(\frac{V_{T}-V_{0}}{\Delta_{T}})$$ (3.12)

$$\frac{1}{\Delta_{T}}(\left(𝓚*\;I^{*}\right)\left(t\right)-\frac{1}{\tau_{m}}\left(V_{0}-E_{L}\right))≔-\left(K-1\right)h_{0}+\frac{d}{dt}\text{ln}\;p_{in}\left(t\right)$$ (3.13)

In other words, the (𝓚-filtered) input current is set linear to $\frac{\text{d}}{\text{d}t}\text{ln}\;p_{in}(t)$. Note that the assumed form of *p*_*in*_(*t*) imposes a constraint on I*(*t*); In particular, if there are no modulations and thus *p*_*in*_(*t*) = *p*_0_(*t*), the resulting current is constant and equal to the current needed to establish constant voltage baseline *V*_0_ (cf. Eq 2.6 when $\dot{V}(t)=0$).

Finally, using $\frac{\text{d}}{\text{d}t}\text{ln}\;p_{in}(t)=-h_{0}+\frac{\text{d}}{\text{d}t}\text{ln}\Delta p_{in}(t)$ and *h*(*t*) = *h*_0_ + Δ*h*(*t*) in Eq 3.13 one ends up with:

$$\begin{array}{ll} \frac{\text{d}}{\text{d}t}\text{ln}\;p_{EIF}\left(t\right)\;\overset{\text{(}2.4\text{)}}{=}\;\frac{\text{d}}{\text{d}t}\text{ln}\;h\left(t\right)-h\left(t\right) & \overset{\text{(}3.11\text{)}}{\approx }\;\left(-Kc_{0}+K-1\right)\Delta h\left(t\right)-h_{0}+\frac{\text{d}}{\text{d}t}\text{ln}\;\Delta p_{in}\left(t\right)\\ & \;=\left(-Kc_{0}+K-1\right)\Delta h\left(t\right)+\frac{\text{d}}{\text{d}t}\text{ln}\;p_{in}\left(t\right) \end{array}$$ (3.14)

Eq 3.14 is the main result of this paper and its significance is as follows: Suppose the term containing Δ*h*(*t*) on the right hand side could be neglected. Then, for small modulations, the EIF neuron would implement the hazard equation of the ideal ISI sampler with $\frac{\text{d}}{\text{d}t}\text{ln}p_{in}(t)$ as input (cf. Eq 2.4). Consequently *p*_*EIF*_(*t*) would be given as the product of exponential baseline *p*_0_(*t*) and the undistorted modulatory term Δ*p*_*EIF*_(*t*) = Δ*p*_*in*_(*t*) and hence *p*_*EIF*_(*t*) = *p*_*in*_(*t*). Because of the derivative, this condition is certainly fulfilled for the high spectral components of ln Δ*p*_*in*_(*t*), regardless of the baseline voltage *V*_0_ that determines *c*_0_. In other words, high frequencies are transduced without distortion from the user-provided input to the ISI output of the EIF neuron.

For low frequencies in contrast, the Δ*h*(*t*) term cannot be neglected, but when *V*_0_ → *V*_*T*_ its weight ∣−*Kc*_0_ + *K* − 1∣ decreases exponentially upon converging to 1. Decreasing this weight term becomes particularly relevant when it is strong, i.e. for large *K*, which is the predominant regime during UP-states, due to the simultaneous presence of high-conductance (i.e. small *τ*_*m*_) and low firing rates ∼ 10Hz (cf. Eq 2.7). This is exactly the proposed mechanism, by which UP-states with baseline levels close to the firing threshold facilitate transduction of the low-frequency components of ln Δ*p*_*in*_(*t*).

For the LIF neuron, steps Eq 3.1 to Eq 3.14 may be repeated in an analogous fashion, upon neglecting the exponential term in Eq 3.3 and substituting the r.h.s. of Eq 3.13 by $h_{0}+\frac{\text{d}}{\text{d}t}\text{ln}\;p_{in}(t)$. Thus, in the LIF case, the input current is also linearly dependent on $\frac{\text{d}}{\text{d}t}\text{ln}\Delta p_{in}(t)$, but with a different y-intercept as for the EIF. The analogous expression of Eq 3.14 then reads:

$$\begin{array}{ll} \frac{\text{d}}{\text{d}t}\text{ln}\;p_{LIF}\left(t\right) & \approx \left(-Kc_{0}-1\right)\Delta h\left(t\right)-h_{0}+\frac{\text{d}}{\text{d}t}\text{ln}\Delta p_{in}\left(t\right)\\ & =\left(-Kc_{0}-1\right)\Delta h\left(t\right)+\frac{\text{d}}{\text{d}t}\text{ln}\;p_{in}\left(t\right) \end{array}$$ (3.15)

Importantly, for *V*_0_ → *V*_*T*_ the weight term ∣−*Kc*_0_ − 1∣ converges to *K* + 1 here, not 1. Hence, unlike the EIF case, Δ*h*(*t*) cannot be neglected for large *K*, even when voltage baseline is high. This means that low-frequency input modulations ln Δ*p*_*in*_(*t*) are severely distorted at the output lnΔ*p*_*LIF*_(*t*).

We will now quantify these arguments in a more rigorous fashion.

### 3.2 Spectral Analysis of the Probability Modulation Transfer Function for the EIF and LIF Neuron

In this section we investigate the degree of distortion, when a log-probability modulation ln Δ*p*_*in*_(*t*) is to be conveyed from the input to the ISI output ln Δ*p*_*EIF*_(*t*), ln Δ*p*_*LIF*_(*t*) of the EIF and LIF neuron respectively. For that, a spectral analysis of the transfer functions from ln Δ*p*_*in*_ to $\text{ln}\Delta p_{\overset{\text{EIF;}}{LIF}}$ is conducted.

From the the definitions of *p*_*EIF*_(*t*) and *p*_*LIF*_(*t*) it follows:

$$\frac{d}{dt}\text{ln}\;p_{0}\left(t\right)=-h_{0}$$ (3.16)

$$\frac{d}{dt}\text{ln}\Delta p_{\overset{\text{EIF;}}{LIF}}\;\left(t\right)=\frac{d}{dt}\text{ln}\;p_{\overset{\text{EIF;}}{LIF}}\;\left(t\right)-\frac{d}{dt}\text{ln}\;p_{0}\;\left(t\right)$$ (3.17)

and hence by Eqs 3.14 and 3.15

$$\frac{d}{dt}\text{ln}\Delta p_{\overset{\text{EIF;}}{LIF}}\;\left(t\right)=K_{\overset{\text{EIF,0;}}{\text{LIF},0}}\;\Delta h\left(t\right)+\frac{d}{dt}\text{ln}\Delta p_{in}\left(t\right)$$ (3.18)

where

$$\begin{array}{ll} K_{EIF,0} & ≔-Kc_{0}+K-1\\ K_{LIF,0} & ≔-Kc_{0}-1 \end{array}$$ (3.19)

Upon Laplace transforming Eq 3.18 the probability modulation transfer functions *T*_*EIF*_(*s*) and *T*_*LIF*_(*s*) may be expressed as (see methods section 2.4 for the derivation):

$$T_{\overset{\text{EIF;}}{LIF}}\;\left(s\right)≔\frac{𝓛\{\text{ln}\Delta p_{EIF;LIF}\left(t\right)\}\left(s\right)}{𝓛\{\text{ln}\Delta p_{in}\left(t\right)\}\left(s\right)}=\frac{s-h_{0}}{s-h_{0}-K_{\overset{\text{EIF,0;}}{\text{LIF},0}}\cdot h_{0}}$$ (3.20)

**Fig 3:**
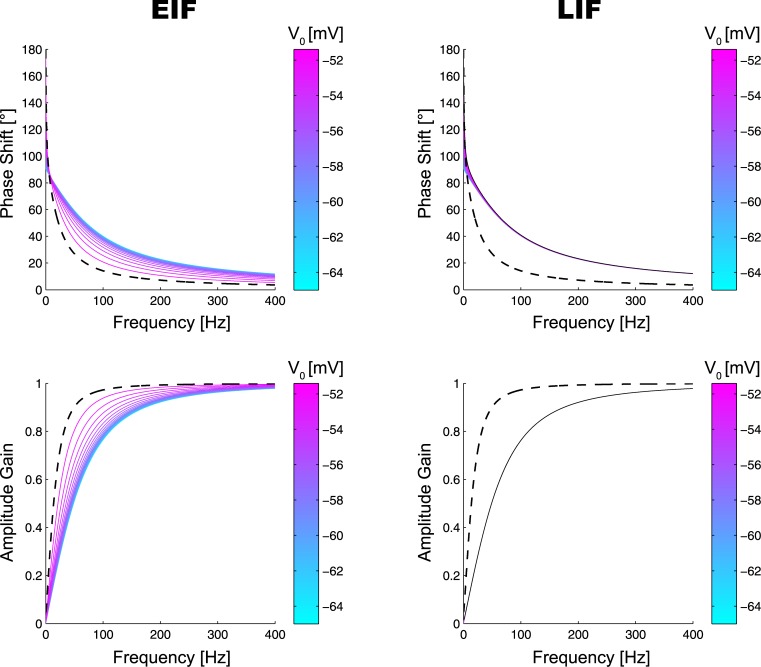
Bode Plots of the EIF and LIF Neuron for Various Different Baseline Voltages *V*_0_ in the High Conductance Regime (*g*_*L*_ = 5·30*nS*, *K* ≈ 53.4). Top row: Phase shift of *T*_*EIF*_(*s*) (left) and *T*_*LIF*_(*s*) (right) as a function of frequency, for baseline voltages *V*_0_ ∈ [−65*mV*, *V*_*T*_ − 1*mV*] (colors). For the EIF neuron, the curve for *V*_0_ = *V*_*T*_ − 1*mV* is drawn as dashed, black line and replotted on the right for comparison. Bottom row: Amplitude gain ∣*T*_*EIF*_(*s*)∣ (left) and ∣*T*_*LIF*_(*s*)∣ (right) for baseline voltages *V*_0_ ∈ [−65*mV*, …, *V*_*T*_ − 1*mV*]. For the EIF neuron, the curve for *V*_0_ = *V*_*T*_ − 1*mV* is drawn as dashed, black line and replotted in the right plot for comparison.

Fig 3 shows the Bode plots of *T*_*EIF*;*LIF*_ (*s*). As expected, when transduced to ln Δ*p*_*EIF*_, the distortion of ln Δ*p*_*in*_ is continuously decreased when *V*_0_ → *V*_*T*_. This is because the higher the baseline *V*_0_, the lower the phase shift (for *f* ≳ 6*Hz*) and the closer the amplitude gain gets to unity. In case of the LIF neuron however the amplitude gain is virtually unaffected by *V*_0_, whereas the phase shift even increases slightly for *V*_0_ → *V*_*T*_.

Importantly, for both measures, phase shift and amplitude gain, performance of the LIF neuron is at most as good as performance of the EIF neuron and corresponds to EIF performance in case of low voltage baselines *V*_0_. This is to be expected, since both neuron models become identical in this voltage regime. Conversely, when *V*_0_ is close to *V*_*T*_ (i.e. when the spiking nonlinearity kicks in) EIF performance continuously improves unlike the LIF neuron.

**Fig 4:**
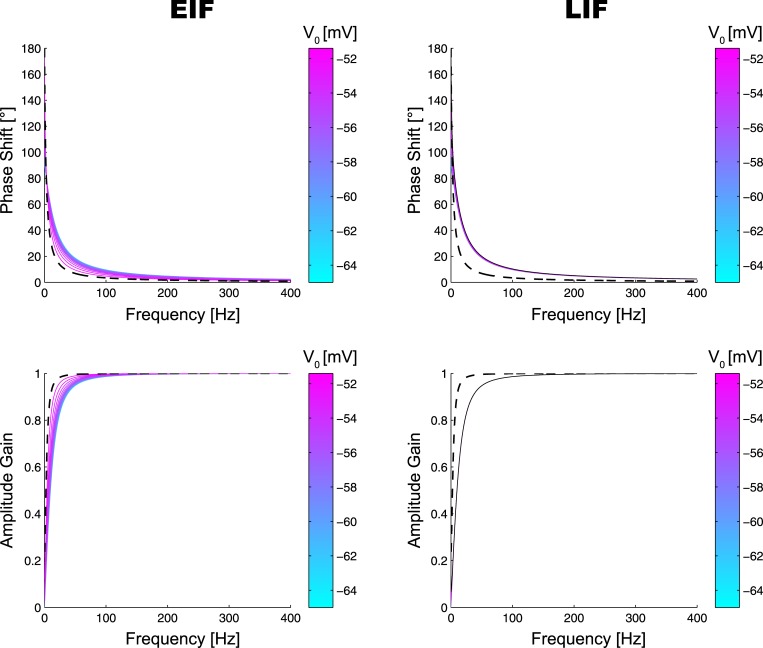
Bode Plots of the EIF and LIF Neuron for Various Different Baseline Voltages *V*_0_ in the Low Conductance Regime (*g*_*L*_ = 30*nS*, *K* ≈ 10.7). See Fig 3 for legend.

Recalling our discussion at the end of the previous chapter, moreover, one expects an increased voltage baseline to become maximally effective in the high conductance regime, where *K* is high as well. A comparison between Figs 3 and 4 shows that this is indeed the case. Although overall performance is better in the low conductance regime, its dependence on *V*_0_ is weaker in the EIF case, both for the phase shift and amplitude gain. Because the high-conductance regime is a necessary byproduct of diffusive noise (due to the large fraction of open synaptic ion channels), our theory thus shows how its deteriorating effects may be overcome by an increased voltage baseline.

To examine the extend to which these theoretical results from small signal analysis hold true in practice, we will now have a look at numerical simulations of both types of neurons and for various settings of *V*_0_ and the probability fluctuation ratio *r*_Δ*p*_.

### 3.3 Performance of the Simulated EIF- and LIF-Neurons for Approximating Predefined ISI Distributions for Various Voltage Baselines and Values of the Probability Fluctuation Ratio

In this section we evaluate the capability of the EIF and LIF neuron to perform random sampling of predefined ISI distributions *p*_*in*_(*t*) during high conductance states. Evaluation is done based on numerical simulations of the two types of neurons, subject to log-modulation functions defined by sums of sinusoids (see methods). First, the neuron’s approximation performances for broadband log-modulation using specific instances of *V*_0_, *h*_0_ and *r*_Δ*p*_ are shown. Then the corresponding summary results are given for sweeps across *V*_0_ and *r*_Δ*p*_.

In order to approximate *p*_*in*_(*t*) by the EIF neuron’s spike output, input current I*(*t*) was computed according to Eq 3.13 (where, in case of the LIF neuron, $-(K-1)h_{0}+\frac{\text{d}}{\text{d}t}\text{ln}\;p_{in}(t)$ was substituted by $h_{0}+\frac{\text{d}}{\text{d}t}\text{ln}\;p_{in}(t)$). For the sake of simplicity we used a simple escape-rate model for stochastic spike-generation, by setting the hazard filter kernel 𝓚 equal to 𝓚(*t*) = *δ*(*t*).

**Fig 5:**
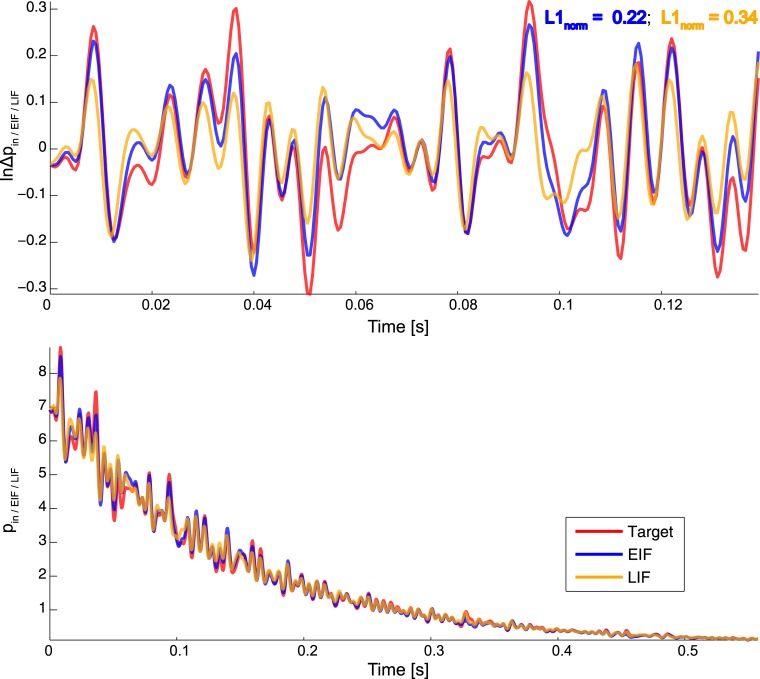
EIF/LIF Performance of Approximating a Predefined ISI Distribution. Top: Log-modulation functions ln Δ*p*_*in*_(*t*), ln Δ*p*_*EIF*_(*t*) and lnΔ*p*_*LIF*_(*t*). Red trace gives the ideal, target log-modulation function ln Δ*p*_*in*_(*t*) (broadband signal consisting of a superposition of 60 sinusoids with random phase, unit amplitude and frequencies taken equidistantly from [10, 200]Hz. Baseline voltage was set to *V*_0_ = −51.4mV, corresponding to a baseline hazard of *h*_0_ = 7.2Hz. Probability fluctuation ratio was set to *r*_Δ*p*_ = 0.44). Blue and orange traces are the actual log-modulation functions ln Δ*p*_*EIF*_(*t*) and lnΔ*p*_*LIF*_(*t*) of the EIF and LIF neuron respectively, obtained by numerical integration (see methods). Bottom: ISI distributions *p*_*in*_(*t*), *p*_*EIF*_(*t*) and *p*_*LIF*_(*t*) corresponding to the modulation functions in the upper plot.

Fig 5 shows an example ISI distribution produced by broadband modulation. As predicted by our theory, there is a better overall match between *p*_*EIF*_ and *p*_*in*_ when compared to the LIF neuron.

Does this finding also hold for a larger range of values for *V*_0_ and *r*_Δ*p*_? To answer that question *V*_0_ was swept across 20 equidistant values between −65mV and *V*_*T*_ − 1mV = −51.4mV (corresponding to a sweep of *h*_0_ between 0.1Hz and 7.2Hz). Likewise *r*_Δ*p*_ was swept across 20 equidistant values between 0.05 and 0.8.

**Fig 6:**
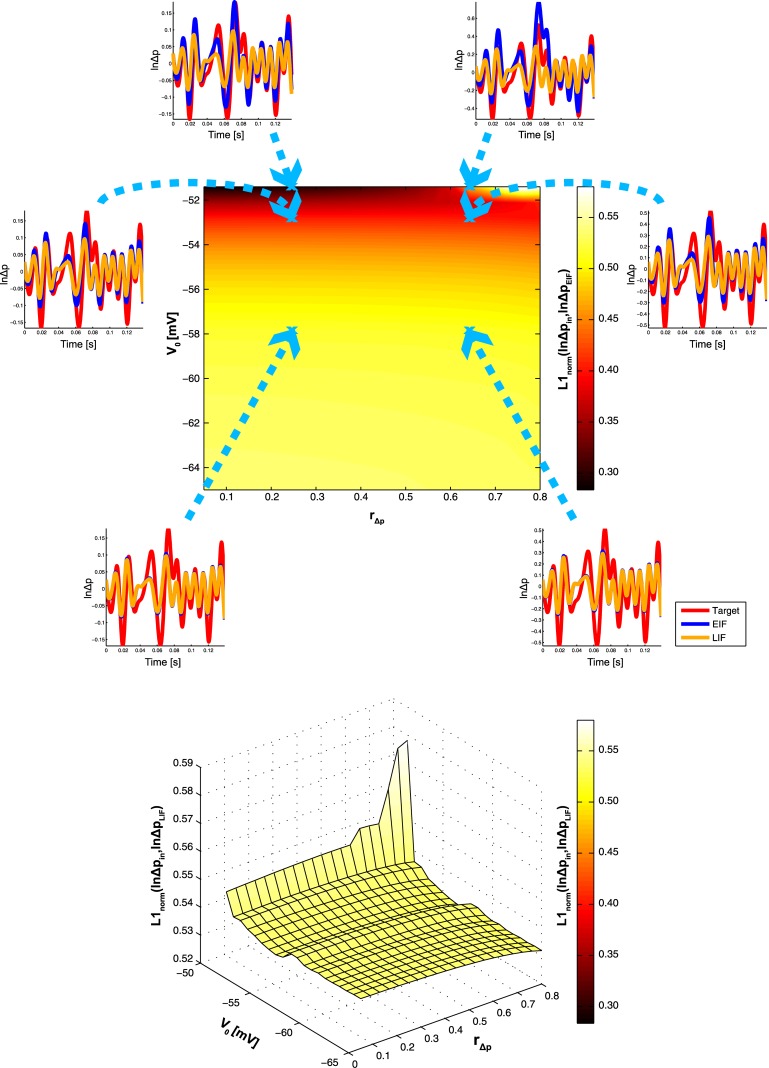
Summary Results of Sweeping *V*_0_ and *r*_Δ*p*_ for Low-Frequency Log-Probability Modulation. a) EIF neuron: Shown is a color-coded contour plot of *L*1_*norm*_(ln Δ*p*_*in*_, ln Δ*p*_*EIF*_) for various combinations of the probability fluctuation ratio *r*_Δ*p*_ (x-axis) and the baseline voltage *V*_0_ (y-axis). The curves are example log-probability modulation functions ln Δ*p*_*in*_ (red) and ln Δ*p*_*EIF*_ (blue). The modulations lnΔ*p*_*LIF*_ of the LIF neuron are also shown for comparison (orange). Values of *r*_Δ*p*_ and *V*_0_ of each example are indicated by blue arrows. b) LIF neuron: Color-coded surface plot of *L*1_*norm*_(ln Δ*p*_*in*_, lnΔ*p*_*LIF*_), color code as in (a).

**Fig 7:**
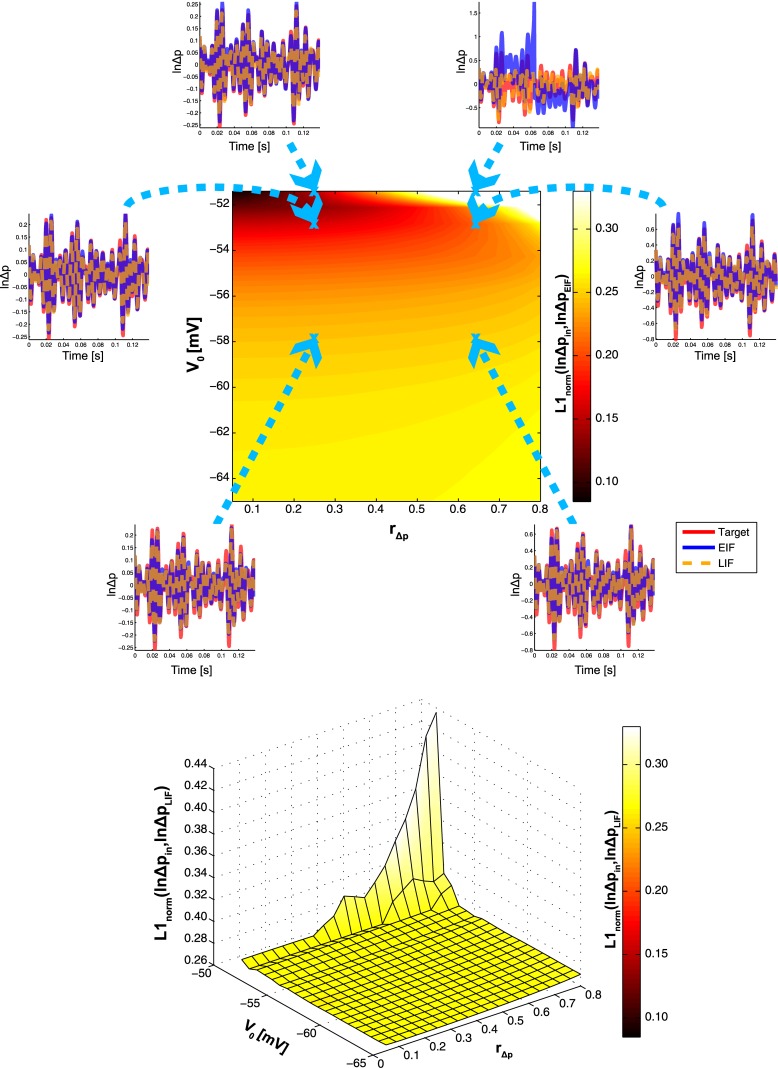
Summary Results of Sweeping *V*_0_ and *r*_Δ*p*_ for High-Frequency Log-Probability Modulation. The same plots as in Fig 6, but for high-frequency modulation.

For each such combination of *V*_0_ and *r*_Δ*p*_, the *L*1_*norm*_(ln Δ*p*_*in*_, ln Δ*p*_*EIF*;*LIF*_) performance measure was evaluated. Note that the sweep covered fairly large values of *r*_Δ*p*_, where results from small signal analysis are not guaranteed to hold. Figs 6 and 7 show the corresponding results for low- and high-frequency modulation respectively. As expected, performance is generally better for high frequencies, both for the EIF and LIF neuron.

In the former case however, there is a dependence of *L*1_*norm*_(ln Δ*p*_*in*_, ln Δ*p*_*EIF*_) on baseline voltage, such that performance increases with increasing *V*_0_, both for low- and high-frequency modulations. It is only for large values of *r*_Δ*p*_ that this effect is attenuated, or even reversed if, in addition, *V*_0_ stays close to the firing threshold. This sharp decrease in performance (which is most obvious in Fig 7) is rooted in the deterministic membrane potential trajectory of the EIF neuron which, for high values of *r*_Δ*p*_, deviates more strongly from the ideal trajectory, as it would manifest itself if EIF dynamics would follow exactly the hazard equation of the ideal ISI sampler. Therefore, there is an inherent danger of erroneously crossing the threshold when situated close to it, which causes strong positive feedback to kick in. Such feedback may then easily amplify any deviation from the ideal trajectory (see e.g. Fig 7, for *V*_0_ = −51.4mV, *r*_Δ*p*_ = 0.64, *t* ≈ 0.065s). Although such a phenomenon cannot be considered an artifact, it is clearly caused by the mechanics of the EIF neuron.

In case of the LIF neuron, erroneous threshold crossings may also occur for high *V*_0_ and *r*_Δ*p*_, but LIF behavior on the remaining parameter space is more stereotypical compared to the EIF neuron: There is virtually no influence of baseline voltage on performance, as the latter hardly changes across the (*r*_Δ*p*_, ln Δ*p*_*LIF*_)-plane, particularly for high-frequency modulations. For low-frequency modulations, there is even a slight decrease in performance when *V*_0_ → *V*_*T*_. Most importantly however, there is no point on the plane where the LIF neuron performs better than the EIF neuron: Both are identical for low *V*_0_ (when the spiking nonlinearity has no influence on the membrane potential), but whereas EIF performance increases with increasing *V*_0_, LIF performance stays constant or even decreases.

Overall, the empirical results presented in this chapter confirm those obtained theoretically from small signal analysis. They are also robust against even large probability modulations, particularly for low-frequencies.

### 3.4 Range of Realizable ISI Distributions

Whereas in the previous sections, the range of distributions our ISI sampling model may sample from was analyzed in the frequency domain, we here estimate this range in terms of the amplitudes of the probability modulation function. This is necessary, because the model is not based on general ISI distributions, but rather on fluctuations around some exponential baseline.

In the following we assume Δ*p*_*EIF*;*LIF*_(*t*) ∼ ln 𝓝(*μ*, *σ* ^2^) to be log-normally distributed for all *t*, i.e. that ln Δ*p*_*EIF*;*LIF*_(*t*) follows some stationary gaussian process with mean *μ* and variance *σ* ^2^. This assumption covers many practically relevant types of probability modulation functions, e.g. all instances of colored, gaussian noise, as they were considered in the previous section. *μ* is set equal to −*σ* ^2^/2, such that the mean 𝔼[Δ*p*] = 1 (to avoid cluttered notation, the subscripts *EIF*;*LIF* of *p*(*t*) are now dropped). At any time *t*, we refer by *p*_*p*_(*t*) ≔ ln 𝓝(*μ* + ln *h*_0_ − *h*_0_ *t*, *σ* ^2^) to the distribution of values of *p*(*t*) = *p*_0_(*t*)Δ*p*(*t*), *p*_0_(*t*) = *h*_0_exp−*h*_0_ *t*. When the probability fluctuation ratio is redefined as the coefficient of variation of Δ*p*(*t*), that is $r_{\Delta p}≔\frac{\sqrt{\text{Var}[\Delta p]}}{𝔼\left[\Delta p\right]}=\sqrt{\text{Var}[\Delta p]}$, then, according to basic properties of the log-normal distribution, the variance *σ* ^2^ may be expressed in terms of *r*_Δ*p*_ by $\sigma^{2}=\text{ln}\;\left(r_{\Delta p}^{2}+1\right)$. Thus, except *h*_0_ from the baseline distribution, *r*_Δ*p*_ remains the only parameter for determining the range of realizable ISI distributions, when log-normal Δ*p*(*t*) are considered.

Because by definition the term Δ*p*(*t*) contains an implicit normalization constant (such that *p*(*t*) is a distribution), it is not straightforward to construct a corresponding gaussian process for lnΔ*p*(*t*), without the use of future information about the process. Fortunately however, the normalization constant is not needed in a realistic, biological setup, due to the derivative employed for computing the neuron’s input current (cf. Eq 3.13). Hence any stationary gaussian process with *μ* = −*σ* ^2^/2 may be considered in our scenario.

**Fig 8:**
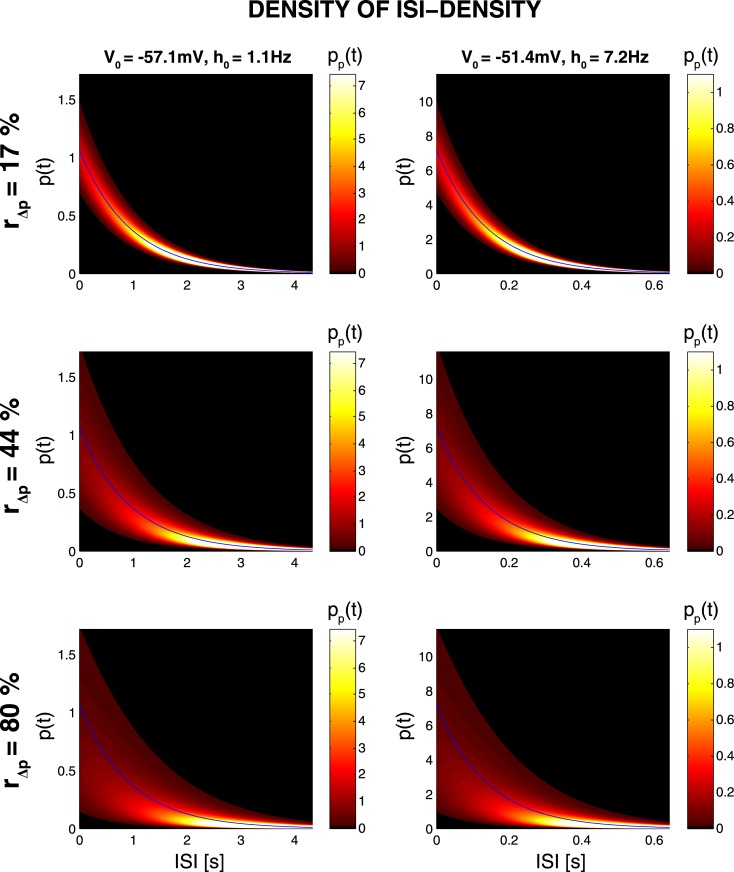
Range of Realizable Values of *p*(*t*) as a Function of Time. Shown are the color-coded distributions *p*_*p*_(*t*) of values *p*(*t*) for different probability fluctuation ratios *r*_Δ*p*_ (rows) and hazard baselines *h*_0_ (columns). For any fixed time *t*, *p*_*p*_(*t*) gives the distribution of values of the ISI distribution *p*(*t*). Blue lines give the baseline ISI distributions *p*_0_(*t*) = *h*_0_ exp(*h*_0_ *t*) that are induced in the absence of probability modulation (*r*_Δ*p*_ = 0).

Fig 8 shows example *p*_*p*_(*t*) resulting from the log-normal model. As expected, *p*_*p*_(*t*) becomes broader for increasing values of *r*_Δ*p*_, i.e. there is a corresponding increase in size of the set of realizable ISI distributions *p*(*t*). The mean and standard deviation of *p*_*p*_(*t*) however decrease monotonically with *t*, indicating a reduced range of *p*(*t*)-values for long ISIs. This behavior is a consequence of the product *p*(*t*) = *p*_0_(*t*)Δ*p*(*t*): Although Δ*p*(*t*) is stationary, the exponentially decreasing factor *p*_0_(*t*) renders the mean and standard deviation of *p*_*p*_(*t*) to approach zero exponentially fast. For the same reason, distributions *p*_*p*_(*t*) which correspond to different hazard baselines *h*_0_ are discriminated only by some scaling factor, with no other qualitative differences in shape, modes etc.

## 4 Discussion

### 4.1 Summary

Using the exponential integrate and fire (EIF) neuron, we have explicitly shown in this paper how the nonlinear sodium current, that is triggered when the membrane potential is situated close to the firing threshold, may facilitate the neuron’s ability to perform random sampling based on interspike-intervals (ISIs). This was shown theoretically, by deriving approximately from the EIF voltage equation the differential equation for the ideal firing hazard, which we termed the ‘hazard equation of the ideal ISI-sampler’ (Eqs 3.14 and 2.4 respectively).

The solution to this approximating equation assumes a particularly simple form, which linearly relates the differentiated logarithm of the ISI density to the convolved and integrated input current (Eq 3.13). This way, the neuron may be regarded as a probability transducer, which receives as input a current-encoded ISI distribution and returns ISI samples from an approximating distribution at the output. Approximation quality depends strongly on the spectral composition of the input distribution: Whereas high-frequency components are displayed distortionless at the output, low-frequencies are distorted in terms of phase-shift and amplitude gain. In the high conductance regime however, both measures can be improved on, by increasing the neuron’s membrane potential baseline towards firing threshold. This effect was derived theoretically and confirmed empirically by concomitant simulations.

Because high conductance, along with a noisy membrane potential balanced close to the firing threshold, is a defining feature of UP-states [28; 30], our results thus demonstrate a clear benefit of UP-states for ISI-based random sampling. We have also shown that improved probability transduction may be attributed directly to the nonlinear sodium current, that distinguishes the EIF from the ordinary leaky integrate and fire (LIF) neuron, because LIF performance never surpassed performance of the EIF neuron in any analytic or simulated scenario.

Finally, because the described effects hold true for multiplicatively modulated exponential distributions at the input of the EIF probability transducer, there is a restriction imposed on the range of ISI distributions the neuron may possibly sample from and we have estimated this range analytically.

### 4.2 Plausibility of the Proposed Model

It may be argued that the range of realizable ISI distributions is too limited for ISI sampling to play a role in neural processing. However, it is possible that during UP states the computational procedures needed, e.g. by the cortex, are restricted to the set of realizable ISI distributions. Moreover, the quasi-exponential envelope of this set (Fig 8) might be a reflection of sampling from log(*ISI*) rather than ISI-distributions [25], such that sampling is possible from a more flexible range of log(*ISI*) distributions, that manifest itself in comparably stereotyped ISI distributions. Interestingly, quasi-exponential ISI distributions are quite common in the cortex (see e.g. [36; 37; 38]) and there is evidence that this might indeed be a reflection of such log(*ISI*)-coding [16; 20]. In particular, they were shown to be most prominent during high-conductance states in a detailed Hodgkin-Huxley type model neuron [39]. ISI distributions may also become more stereotyped (i.e. quasi-exponential) through the averaging of nonstationary distributions; Suppose, as in section 3.4 for example, a probability modulation function Δ*p*(*t*) fluctuating around 1. If these modulations are not influenced in any way by their induced spikes (i.e. if the resulting spike train is not renewal), then the (momentary) ISI distribution *p*(*t*) = *p*_0_(*t*)Δ*p*(*t*), *p*_0_(*t*) = *h*_0_ exp(−*h*_0_ *t*) will be slightly different after each such spike, due to the fluctuations of Δ*p*(*t*). Reflecting a situation typically encountered in experiments, the ISI distribution as it is measured from a complete train of spikes will thus be given as the stereotyped average distribution *p*_0_(*t*) and hence be exponential (blue lines in Fig 8).

Another objection against our model is based on the fact that the neuronal simulations of section 3.3 could have been arranged to produce even exact results with zero *L*1-norm error. For that, a distribution representing input current *I*(*t*) can be computed from the user-defined ISI distribution *p*(*t*) via the sequence *p*(*t*) → *h*(*t*) → *V*(*t*) → *I*(*t*) (corresponding to the sequence of insertions into Eqs (2.2) → (2.3) → (2.7) → (2.5)). Although such a procedure is feasible even for the LIF neuron [40; 25], compared to Eq 3.13 it yields a tremendously more complicated expression for the relationship between *I*(*t*) and *p*(*t*). In particular, the transition *p*(*t*) → *h*(*t*) requires computation of the survivor function for which, in a realistic scenario, the set of neurons providing *I*(*t*) must explicitly represent the running integral $\int_{0}^{t}p(t^{\prime })\text{d}t^{\prime }$, e.g. by means of some chemical accumulation variable. This requirement remains, even if the necessary computations are thought to happen as a combination of synaptic inputs and local information processing inside the sampling neuron. In contrast, the approach put forward in this paper naturally circumvents this problem; In case of escape noise, that is for 𝓚(*t*) = *δ*(*t*), the total input current *I*(*t*) directly encodes the momentary value of $\frac{\text{d}}{\text{d}t}\text{ln}\;p(t)$ (ref. Eq 3.13). Even for general filter kernels 𝓚(*t*) however, computation of $\frac{\text{d}}{\text{d}t}\text{ln}\;p(t)$ just requires a static, linear filter applied to the total current and not some highly nonlinear processing applied to an accumulation variable with reset mechanism.

On the other hand, it is questionable whether escape noise is a realistic model of noise in biological neurons. In fact, measurements in cortex during slow wave sleep, where UP/DOWN state transitions are prominent, show that during UP states diffusive noise is present [5; 8] and a crucial factor for spiking variability [5; 10]. Although for the LIF neuron diffusive noise, which is generated by barrages of balanced excitatory and inhibitory synaptic inputs [27], can be approximated to a good degree by escape noise [35], an analogous model for the EIF neuron with a voltage baseline close to threshold is still missing. However, as we have shown in section 3.1, the EIF approximation of the ideal ISI-sampler is still valid for general convolution kernels 𝓚(*t*). Hence, should the logarithm of the hazard, caused by diffusive noise in the EIF neuron, be expressible as a convolution between 𝓚(*t*) and *V*(*t*) (ref. Eq 2.7), our theory remains valid, even for diffusive noise. To establish this requirement, the methods presented in [41] could prove useful. In this context it is also interesting to note that the EIF sampling model provides a clear reasoning why measurements during cortical UP-states have revealed simultaneous occurrence of a high subthreshold voltage baseline, high conductance and high noise levels [8; 10]. Whereas previous explanations attributed this phenomenon to stochastic resonance [27], it is the obtaining of a better approximation of the ideal ISI-sampler, together with the need of a noise source for random firing, that our theory postulates.

With respect to our scenario of voltage baselines close to threshold, one could argue that under sustained depolarizations, neurons tend to increase their threshold, thereby leaving the range where sodium currents could possibly exert an influence on subthreshold processing. Indeed, such a threshold shift has been observed in experiments and can be explained by the sodium inactivation variable from the Hodgkin-Huxley formalism [42]. Because the EIF model is derived from the more detailed Hodgkin-Huxley model by assuming constant inactivation (see [43], chapter 5), this effect appears to be a potential threat to our theory. Experimental data shows however that the shift in threshold is substantially smaller than its causing depolarization (see [42], Fig 2A). This may also explain the apparent consensus in the literature that (high-conductance) UP-states lead to closer proximity of the baseline membrane potential to threshold [39; 8; 44].

**Fig 9:**
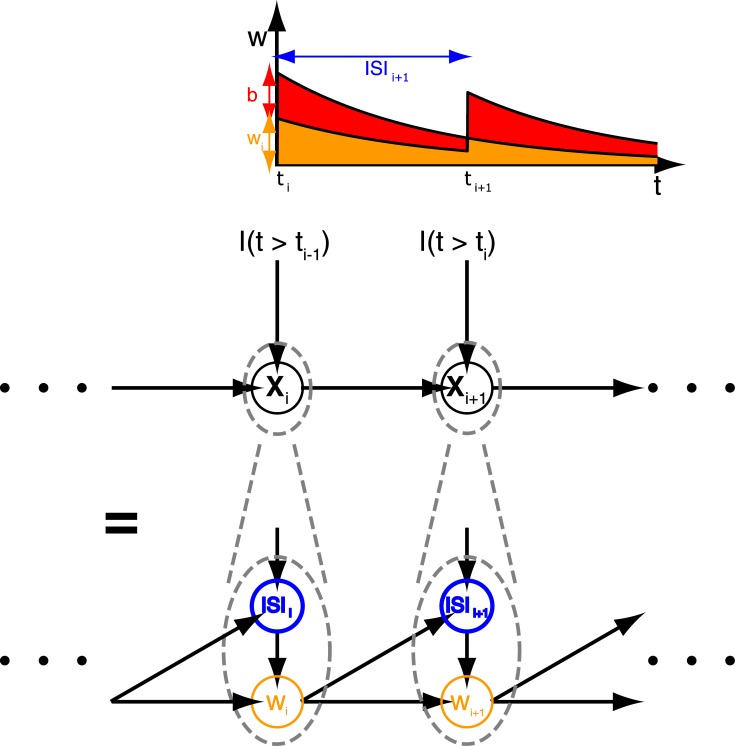
ISI Sequences of the AdEx Neuron Correspond to Sequences of Random Samples from a Markov Chain. Top: Illustration of the spike-triggered dynamics of adaptation current *w* (Eq 4.1), when voltage fluctuations Δ*V*(*t*) can be neglected. Each spike, sequenced by *i*, leads to an immediate increase of *w* by some fixed amount *b*. Between spikes, the dynamics of *w* are governed by a leaky integrator. *w*_*i*_ is the value of *w* immediately before the spike, i.e. before the addition of *b*. *ISI*_*i*_ is the ISI label of the *i*-th spike. Middle: Combining *ISI*_*i*_ and *w*_*i*_ yields a state vector **x**_*i*_ ≔ (*ISI*_*i*_, *w*_*i*_) that follows Markov dynamics. Shown is a Bayesian network representation of the resulting Markov chain. Input to the chain is given by an input current *I*(*t*) provided by the neuron’s presynaptic partners. Bottom: Detailed Bayesian network when **x**_*i*_ is expanded into its components *ISI*_*i*_ and *w*_*i*_. The dependencies shown as arrows are due to Eq 3.13 and the leaky integrator dynamics governing *w*(*t*) (Eq 4.1).

Another objection against the proposed EIF sampling model is its neglect of spike-frequency adaptation. In fact, coupling the EIF dynamics Eq 2.5 with the following equation describing adaptation yields the AdEx model [31], which was found able to reproduce a large range of electrophysiological firing patterns, such as adaptation, bursting, fast and regular spiking:

$$\tau_{w}\frac{dw(t)}{dt}=a\left(V\left(t\right)-E_{L}\right)-w\left(t\right)$$ (4.1)

where *w* is an adaptation current that is subtracted from the right hand side of Eq 2.5 and thus tends to hyperpolarize the membrane and consequently to decrease the firing rate of the neuron. *a* is an subthreshold adaptation parameter, which models the dependence of *w* on membrane voltage *V*. In addition to Eq 4.1, adaptation dynamics are governed by the instantaneous increase of *w* by an amount *b*, each time the neuron fires a spike (Fig 9, top). This rather phenomenological model of adaptation (which subtracts a state-dependent current from the neuron’s input) was found able to match with great accuracy the spike times and voltage evolution of a detailed Hodgkin-Huxley (HH) type model neuron, that contained a biophysically plausible muscarinic potassium current as mechanism for adaptation [31; 45]. Importantly however, the impact of adaptation *fluctuations*, that is, *w*(*t*) subtracted by its average, on the ability of the AdEx to reproduce such spiking patterns was found to be weakest during high conductance states [31]. Thus, even in presence of adaptation, it may be possible to sample from ‘user-defined’ input distributions that are state-*in*dependent of the sampling neuron. This relative unimportance of adaptation dynamics during UP-states is also in line with the apparent independence between two small, subsequent ISIs in mouse somatosensory neurons (see [32], Fig 3A), since dependence between subsequent ISIs is plausibly mediated by spike-frequency adaptation [46; 47].

On the other hand, adaptation may even be incorporated as a computational feature by our ISI sampling scheme: Suppose the adaptation dynamics Eq 4.1 were independent of voltage fluctuations, such that *w* is turned into a leaky integrator. That is, *w* is governed only by the output spike train, voltage baseline *V*_0_ and adaptation parameters *a* and *b*, whereas voltage fluctuations due to Δ*V*(*t*) are neglected (Fig 9,top). This assumption is plausible in our considered scenario, because of the small voltage fluctuations the ISI sampling model is based on (cf. assumption (a) in section 3.1). In this case, if we define **x**_*i*_ ≔ (*ISI*_*i*_, *w*_*i*_) as a state vector, consisting of an ISI-label *ISI*_*i*_ (given by the *i*-th spike in a train) and *w*_*i*_ (the value of *w* immediately before the *i*th spike), then the sequence of ISIs put out by the AdEx neuron corresponds to a sequence of samples from a Markov chain (Fig 9, middle and bottom). As before, external input to the Markov chain is provided by *I*(*t*), note however that here the total input current of the neuron is given by *I*(*t*) − *w*(*t*). For the future, we have plans to further explore the computational implications imposed by this biologically more realistic setup.

### 4.3 Computational Interpretations of ISI-Based Random Sampling During UP-States

Random sampling also fits seamlessly into the long standing hypothesis of oscillating UP and DOWN states as a means of memory consolidation during slow wave sleep. For example, based on the wake-sleep algorithm [48] (WSA, the notion of ‘sleep’ here is different from actual sleep as in slow-wave sleep) Destexhe and Sejnowski pushed forward a qualitative version of such a theory, that utilizes self-generated, idealized top-down inputs for learning the feedforward connections of an internal object recognition model [12; 13]. Learning such a recognition model is a crucial subroutine of the WSA, whose actual goal however is to learn a generative model, that is a model of the probability distribution of data inputs [48]. The latter model is termed ‘generative’, because it is used by the WSA to generate samples from the distribution the model represents, i.e. fantasized top-down sensory inputs that can be interpreted as memories and which are used for updating the recognition model during the so called sleep phase of the algorithm.

One interpretation of the alternating sequence of UP and DOWN states during slow wave sleep could be the mimicking of such a sleep phase of the WSA. That is, during UP states, ISI samples are drawn from the generative model and their values are stored by the sampling neuron. During the subsequent DOWN state, these values are then used by the neuron to update the parameters that control the density of the recognition model. Based on these updated parameters, a new set of samples is drawn in the next UP state and the procedure repeats, thereby mimicking a version of stochastic gradient ascent [49], as it is used by some formulations of the WSA [48]. If each neuron represents one dimension of some high-dimensional generative model, synaptic connections are necessary to mutually influence the sampling process performed by each neuron. Such influencing by exchanging sampled values is crucial for sampling in high-dimensional spaces and is a hallmark of MCMC-sampling methods such as Gibbs-sampling [22; 24]. The presented computational theory also explains the experimentally observed synchrony of UP/DOWN state transitions across cortical neurons [4; 8; 50; 44]. During an UP state, each neuron must be guaranteed to have its sampling based on the same high-dimensional distribution, such that procedures like Gibbs-sampling become feasible. This is achieved by restricting the windows of opportunity for parameter updates to synchronous DOWN states. In this context, it is also interesting to note that recent experiments have indicated ongoing cortical activity during UP states to be functionally protected from thalamic inputs [44]. For the same reason this is exactly what is to be expected, if the goal of the UP-state is indeed to perform MCMC sampling from an unperturbed (prior) generative distribution that is represented in the sensory deprived cortex.

Similarly, a related interpretation of synchronous UP/DOWN state transitions is based on the EM-algorithm [51]. In this case, UP states would correspond to expectation (E) steps, during which sampling-based inferences about hidden variables in the generative model are conducted. As with the WSA, the subsequent maximization (M) steps would then be confined to updates of the parameters.

### 4.4 Model Predictions

As indicated in the section 4.2, our model hinges on the validity of Eq 2.7 for describing the dependency between membrane voltage and firing hazard. In particular, Eq 2.7 predicts the spiking determinism parameter to be equal to Δ_*T*_, the slope factor of the EIF model. In this context, it is interesting to note that there is indeed evidence for an exponential escape-rate model to be a good empirical descriptor of the hazard near firing threshold (in case of an EIF neuron stimulated by diffusive noise) [41]. Also, a quite remarkable congruence between the spiking determinism parameter and Δ_*T*_ has been reported in the literature. More specifically, reported values for Δ_*T*_ are from the set {0.5, 1.4, 3, 3.48}mV [52; 31; 53], whereas reported values for the spiking determinism parameter are {0.5, 3, 4}mV [54; 55].

Therefore, if Eq 2.7 turns out indeed to be a sensible model, e.g. for cortical neurons during UP states *in vivo*, then our theory could be tested further, for example by repeatedly injecting traces of current into a cortical neuron and examining the resulting ISI distribution. More specifically, triggered by each spike of the neuron, a current profile computed from Eq 3.13 could be injected and the ISI distribution be compared to the predicted one according to Eq 3.14.

In contrast, if in experiments the proposed dependency between membrane voltage and hazard turns out *not* to be a good description, then this aspect serves as a means to falsify the whole theory relating ISI sampling to EIF processing near threshold.
